# Retinal Biomarkers of Folate and Vitamin B12 Metabolic Dysfunction: A Framework for Machine Learning-Assisted Detection of Cerebral Folate Deficiency

**DOI:** 10.3390/cells15161453

**Published:** 2026-08-13

**Authors:** George Ayoub, Craig Brown

**Affiliations:** 1 Psychology Department, Santa Barbara City College, Santa Barbara, CA 93109, USA; 2 Department of Ophthalmology, University of Arkansas for Medical Sciences, Fayetteville, AR 72703, USA; cjbrown2@uams.edu

**Keywords:** cerebral folate deficiency, retinal biomarkers, machine learning, optical coherence tomography, OCTA, vitamin B12 deficiency, MTHFR, hyperhomocysteinemia, folate receptor autoantibodies

## Abstract

**Highlights:**

**What are the main findings?**

- Folate/B12 metabolic dysfunction (nutritional deficiency, FRα autoantibodies, MTHFR/DHFR variants) produces convergent, quantifiable retinal changes that are detectable with OCT/OCTA and often reversible with early correction.
- The review proposes a conceptual multimodal ML framework, combining retinal imaging with biochemical and genetic data, as a research agenda for non-invasive detection of cerebral folate deficiency (CFD).

**What are the implications of the main findings?**

- A validated retinal-imaging/ML approach could enable non-invasive prenatal and neonatal screening for CFD in FRAA-positive or genetically high-risk pregnancies, allowing earlier leucovorin treatment.
- Given the folate–ASD risk association reported in FRAA-positive cohorts, earlier identification and treatment of maternal/infant CFD may plausibly reduce neurodevelopmental risk, including autism spectrum disorder, in offspring.

**Abstract:**

Disruptions in folate (vitamin B9) and vitamin B12 metabolism, including nutritional deficiency, folate receptor alpha (FRα) autoantibodies, and MTHFR/DHFR polymorphisms, impair one-carbon metabolism and produce measurable retinal structural, microvascular, and functional changes, offering a non-invasive window into systemic and cerebral metabolic dysfunction, particularly cerebral folate deficiency (CFD). This review synthesizes peer-reviewed evidence on retinal alterations linked to folate/B12 deficiency, hyperhomocysteinemia, FRα autoantibody syndromes, and MTHFR/DHFR variants, alongside artificial intelligence (AI) and machine learning (ML) approaches applied to retinal imaging for metabolic, anemic, and nutritional optic neuropathy detection. Three convergent phenotypes emerge: structural changes (retinal nerve fiber layer and ganglion cell complex thinning, optic disc pallor, chorioretinal atrophy), microvascular abnormalities (reduced vessel density, foveal avascular zone enlargement, capillary dropout), and functional deficits (centrocecal scotoma, dyschromatopsia, reduced contrast sensitivity); they arise from homocysteine-mediated endothelial toxicity, mitochondrial impairment, and eNOS uncoupling. Existing AI/ML models for anemia and optic neuropathy establish technical feasibility but do not target folate-specific phenotypes. We propose a dedicated multimodal ML framework integrating structural, perfusion, functional, and biochemical/genetic data as a research agenda for automated, non-invasive CFD detection. Given the established folate–autism spectrum disorder (ASD) risk association reported in FRAA-positive cohorts, such a framework, once validated, could support prenatal and neonatal screening in FRAA-positive or genetically high-risk pregnancies, enabling earlier leucovorin treatment and reducing neurodevelopmental risk.

## 1. Introduction

Folate (vitamin B9) is an indispensable cofactor in one-carbon metabolism, mediating DNA synthesis and repair, nucleotide biosynthesis, and the methylation of proteins, lipids, and nucleic acids. Within mitochondria, folate-dependent reactions support oxidative phosphorylation through the formate shuttle and tetrahydrofolate (THF)-linked pathways. Vitamin B12 (cobalamin) operates as an essential cofactor in the same metabolic network, participating in the conversion of homocysteine to methionine (methionine synthase) and methylmalonyl-CoA to succinyl-CoA (methylmalonyl-CoA mutase). The interdependence of these vitamins means that deficiency or dysfunction of either produces overlapping clinical and biochemical consequences, including hyperhomocysteinemia, impaired methylating capacity, and mitochondrial dysfunction [1,2].

The retina, a metabolically demanding outpost of the central nervous system [3], is particularly vulnerable to these disruptions, as one-carbon (1C) metabolism is critical for continuous photoreceptor renewal, retinal vascular homeostasis, and the control of oxidative stress through efficient homocysteine recycling [4,5,6,7]. Although the eye is a compact organ, it possesses an exceptionally high energy demand [8], and retinal ganglion cells (RGCs), especially those of the maculopapular bundle projecting to the fovea and optic disc, have exceptional mitochondrial density and energy requirements. Disrupted folate or B12 metabolism impairs axonal energy supply, promotes oxidative stress, and compromises vascular endothelial integrity, leading to measurable structural, perfusion, and functional changes detectable with modern ophthalmic imaging [2,9].

Disturbed 1C metabolism has been implicated in each of the three most prevalent causes of irreversible vision loss worldwide: age-related macular degeneration (AMD), glaucoma, and diabetic retinopathy. Elevated homocysteine, a hallmark of impaired 1C flux and a well-recognized systemic disease biomarker, is associated with microvascular dysfunction, breakdown of the blood–retinal barrier, and increased risk of diabetic retinopathy and AMD, positioning 1C metabolites as both biomarkers and mechanistic contributors to disease [10,11]. In glaucoma, metabolomic and genetic data indicate that 1C pathways are dysregulated, and correcting these defects in experimental models attenuates retinal ganglion cell loss and optic nerve damage. Collectively, these observations support a model in which defects in folate-mediated and methionine cycle-dependent reactions converge on oxidative injury, mitochondrial stress, and neurovascular instability across these seemingly distinct diseases [5,6,12,13,14].

Because 1C metabolism is nutrient-dependent, it represents a modifiable therapeutic target. Folate, vitamins B2, B6 and B12, and choline are indispensable cofactors for homocysteine remethylation and transsulfuration, and their deficiency is a leading cause of elevated homocysteine and impaired methylation capacity. Experimental supplementation with these vitamins in animal models of glaucoma normalizes 1C flux, reduces homocysteine, and preserves retinal structure and optic nerve function, while B-vitamin repletion in hyperhomocysteinemic models of retinal vascular disease improves barrier integrity and mitigates neurovascular damage. Observational and interventional data in humans further suggest that higher intake or supplementation of B vitamins, including folate, may reduce the risk or slow progression of diabetic retinopathy, AMD, and glaucoma, though definitive randomized evidence is still emerging [6,7,12,13,14,15,16].

Beyond simple nutritional deficiencies, disruptions in folate metabolism encompass a spectrum of etiologies: (1) dietary or malabsorptive folate deficiency; (2) folate receptor alpha (FRα) autoantibody-mediated transport blockade causing cerebral folate deficiency (CFD); (3) polymorphisms in methylenetetrahydrofolate reductase (MTHFR, variants C677T and A1298C) and dihydrofolate reductase (DHFR, 19 bp deletion) that reduce enzymatic efficiency; and (4) overlapping deficiencies in vitamins B2 (riboflavin) and B6 (pyridoxine), which share the homocysteine remethylation and transsulfuration pathways [2,4,7,17,18,19]. Each etiology contributes to a partially overlapping retinal phenotype.

**Figure 1:**
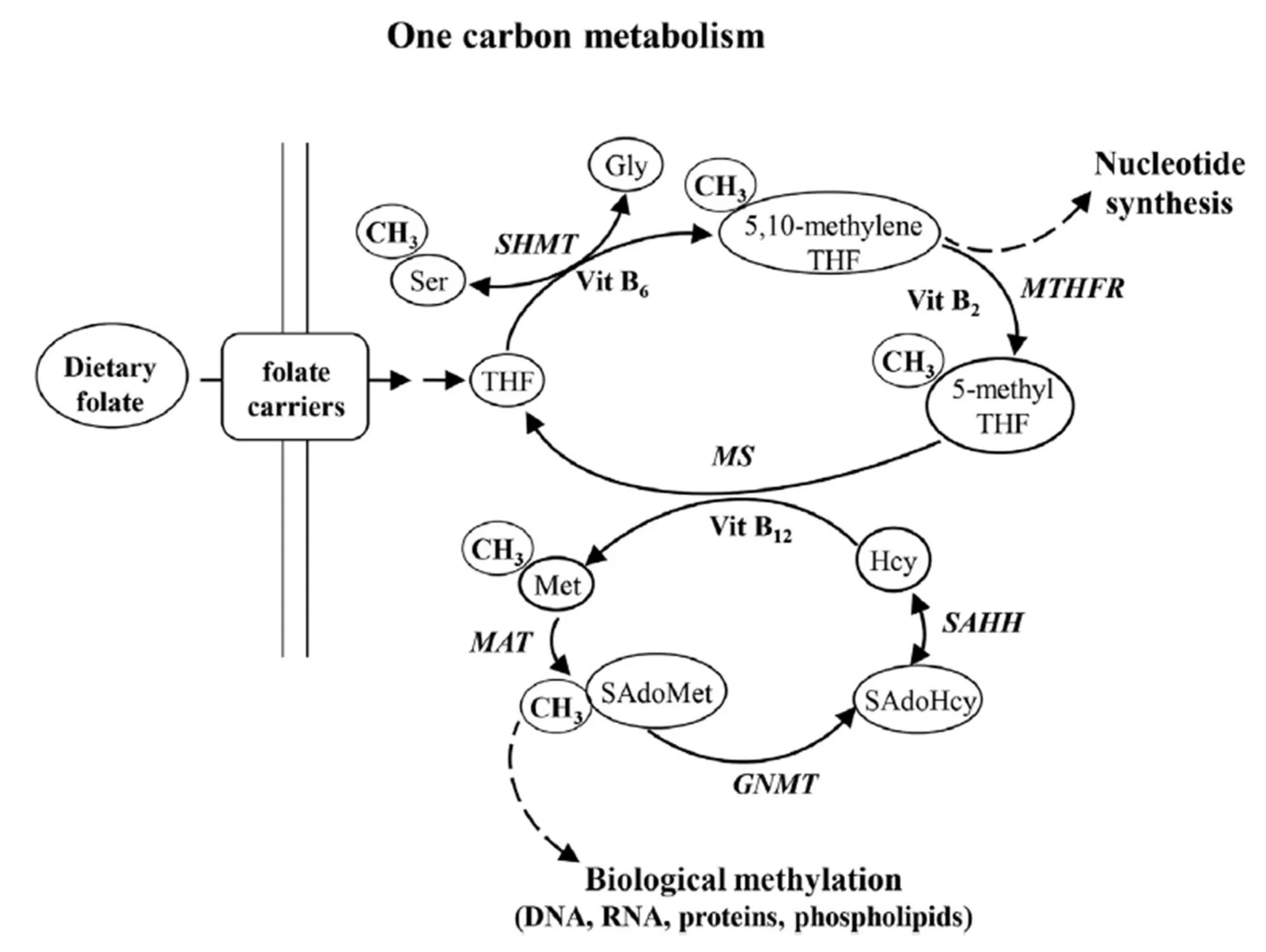
Nutrients involved in one-carbon metabolism [7,23].

Mechanistically, folate acts as a carrier of one-carbon units: driven by serine hydroxymethyltransferase (SHMT) and its cofactor vitamin B6, THF accepts a one-carbon unit from serine to form 5,10-methylenetetrahydrofolate (5,10-methylene-THF) [20]. A portion of this intermediate is used directly as a substrate for thymidylate (dTMP) synthesis or is oxidized to 10-formyltetrahydrofolate (10-formyl-THF), the sole donor for purine synthesis, while the remainder is converted by MTHFR into 5-methyltetrahydrofolate (5-MTHF), the principal dietary folate and methyl donor for the remethylation of homocysteine to methionine and, ultimately, for SAM-dependent methylation. Because 10-formyl-THF sustains the nucleotide pool required for the vigorous DNA repair activity of photoreceptors, folate deficiency compromises both the methylation and nucleotide-synthesis arms of retinal metabolism simultaneously. This THF-dependent network is compartmentalized across the cytoplasm, mitochondria, and nucleus, with mitochondria supplying a substantial share of the one-carbon units used in cytoplasmic reactions via formate release, directly linking mitochondrial energetics to nuclear methylation and nucleotide synthesis (Figure 1) [7,21,22,23].

These reactions are equally dependent on the remaining B vitamins, and genetic polymorphisms such as those affecting MTHFR, combined with B-vitamin deficiencies, can disrupt 1C metabolic homeostasis, driving excessive accumulation of toxic homocysteine that compromises the structural and functional integrity of vascular endothelial cells and the blood–retinal barrier [24,25]. Vitamin B6, in its active coenzyme form pyridoxal-5′-phosphate, is required both for SHMT-mediated 1C mobilization and for the transsulfuration of homocysteine to cystathionine, a precursor of the antioxidant glutathione; B6 deficiency therefore restricts 5-MTHF synthesis while simultaneously impairing homocysteine clearance [7,26,27]. Vitamin B12 (cobalamin) is the obligate cofactor for methionine synthase, which transfers the methyl group from 5-MTHF to homocysteine; when B12 is deficient, this remethylation step stalls, folate becomes metabolically trapped as 5-MTHF (the “methyl trap”), and homocysteine accumulates despite otherwise adequate total folate levels, ultimately manifesting clinically as megaloblastic anemia when nucleotide synthesis fails outright [7,28,29,30].

Beyond its role in these retinal diseases, the 1C cycle is also essential to endothelial cell patency, and its disruption alters blood flow and perfusion throughout the vascular tree [6,24]. Folate and B12 are therefore central not only to the specific retinal diseases discussed above but to the maintenance of healthy retinal vasculature more broadly. Critically, this vascular dependence can be leveraged diagnostically: folate crosses the blood–retinal barrier largely via the same transporters, including folate receptor alpha and the reduced folate carrier, that mediate its passage across the blood–brain barrier; thus, retinal folate transport parallels cerebral folate transport [4,31,32]. Retinal vasculature is embryonically derived from brain vasculature; it therefore offers an accessible, non-invasive window onto a CNS transport system otherwise inaccessible in vivo, providing a rationale for using retinal vascular imaging to identify cerebral folate deficiency (CFD).

Concurrently, advances in ophthalmic imaging, particularly spectral-domain and swept-source OCT (SD/SS-OCT) for structural analysis and OCTA for capillary-level perfusion mapping, have provided quantitative, reproducible metrics capable of detecting subtle retinal changes before symptomatic vision loss occurs [33,34]. Artificial intelligence and deep learning systems have demonstrated high performance in identifying retinal signatures of anemia, nutritional optic neuropathies, and microvascular disease [35,36,37].

The clinical motivation for developing such a system is clear: a rapid, non-invasive means of identifying CFD would allow prompt initiation of targeted treatment with leucovorin (folinic acid), bypassing FRα-mediated transport blockade and restoring cerebral folate status [17,38]. Because CFD and FRα autoantibodies are mechanistically implicated in ASD, identifying and treating maternal or infant CFD earlier, whether prenatally in high-risk pregnancies or in early childhood, carries potential to reduce the incidence of autism spectrum disorder and to ease neurological symptoms in children already diagnosed with the condition [38,39].

The argument developed in this review rests on a chain of inferential steps whose evidentiary strength differs considerably, and we make that chain explicit here. (1) Folate/B12 metabolic dysfunction produces measurable retinal structural, microvascular, and functional changes. This is well established, and documented across multiple cohorts and etiologies (nutritional deficiency, FRAA syndromes, MTHFR/DHFR variants). (2) Retinal folate transport parallels cerebral folate transport. This is biologically plausible, motivated by shared transporters (FRα, the reduced folate carrier) and the shared embryological origin of retinal and cerebral vasculature [4,31,32], but shared transport machinery does not by itself demonstrate that a retinal readout tracks cerebral folate status quantitatively; no study has directly correlated retinal imaging metrics with CSF 5-MTHF or other direct measures of CFD. (3) Retinal imaging can therefore be used to infer CFD. This step is conjectural, extrapolated from (1) and (2) rather than directly demonstrated. (4) CFD/FRα autoantibody status is associated with ASD. This is reasonably well supported in FRAA-positive cohorts, where FRAA prevalence is markedly elevated relative to the general population [38], though the relationship is observational, and the causal contribution of CFD specifically (as opposed to correlated factors) to ASD risk is not fully resolved. (5) Retinal ML screening could therefore support prenatal ASD risk stratification. This is the most speculative link in the chain, compounding the uncertainty in steps (2)–(4), and should be read as a proposed research direction rather than a validated or near-term clinical application. We flag this chain explicitly so readers can distinguish which components of our proposal rest on established evidence and which rest on plausible but unproven extrapolation.

This report has two primary objectives: (1) to synthesize the peer-reviewed evidence on retinal structural, microvascular, and functional changes associated with folate and B12 metabolic dysfunction; and (2) to propose a conceptual framework, grounded in the shared vascular transport biology of the retina and brain, for an ML-assisted system capable of detecting CFD from multimodal retinal imaging, an approach with potential clinical applications ranging from individual patient diagnosis to prenatal risk stratification for neurodevelopmental disorders including autism spectrum disorder (ASD) [38,39].

## 2. Methods

### 2.1. Literature Search Strategy

A search of PubMed/MEDLINE, PubMed Central (PMC), Investigative Ophthalmology & Visual Science (IOVS), Cochrane Database, and Google Scholar was conducted through June 2026. Search terms included combinations of: “folate deficiency,” “vitamin B12 deficiency,” “cobalamin deficiency,” “nutritional optic neuropathy,” “cerebral folate deficiency,” “folate receptor autoantibodies,” “MTHFR polymorphism,” “DHFR polymorphism,” “hyperhomocysteinemia,” “retinal nerve fiber layer,” “RNFL,” “ganglion cell complex,” “OCT,” “OCTA,” “machine learning optic neuropathy,” and “artificial intelligence fundus.” Further relevant papers were sought using Grok AI in hopes it may find references outside the databases used. Reference lists of retrieved articles were manually searched for additional relevant studies.

The Supplemental Materials has a table (Table S1) that summarizes 32 published reports naming some aspect of AI or machine learning to diagnose autism. We were unable to find any published reporting that uses machine learning to identify folate or vitamin B12 deficiency that can result in cerebral folate deficiency, a correctable condition that contributes to autism development [40] and symptoms [41,42]. The only ASD identification study using machine learning we found is one published report using color fundus images to identify ASD from neurotypical children [43], however they did not report the folate status of these children.

### 2.2. Inclusion and Exclusion Criteria

Studies were included if they: (1) reported quantitative or qualitative retinal changes in human subjects, animal models, or cell culture systems relevant to folate or B12 metabolic dysfunction; (2) used OCT, OCTA, fundus photography, fluorescein angiography (FA), microperimetry, visual field testing, electrophysiology, or functional vision testing; or (3) described AI/ML systems applied to retinal imaging for nutritional deficiency, anemia, or optic neuropathy detection. Studies were excluded if they addressed retinal disease unrelated to B-vitamin metabolism or provided insufficient methodological detail. Case reports were included when they provided unique phenotypic characterization.

### 2.3. Data Extraction and Quality Assessment

Data were extracted on study design, sample characteristics, imaging modalities, quantitative metrics reported (e.g., RNFL thickness, vessel density, visual acuity), and clinical outcomes. Given the heterogeneous study designs, ranging from randomized trials to case reports, formal meta-analysis was not feasible; instead, narrative synthesis was performed, with evidence quality assessed using the Oxford Centre for Evidence-Based Medicine levels of evidence framework.

## 3. Pathophysiological Mechanisms of Retinal Injury

The retinal changes associated with folate and B12 metabolic dysfunction converge on four principal pathophysiological axes, which are not mutually exclusive and frequently co-occur in clinical and experimental settings.

### 3.1. Hyperhomocysteinemia and Endothelial Toxicity

The most consistently documented mechanism linking folate and B12 deficiency to retinal injury is hyperhomocysteinemia (HHcy). Both vitamins are essential for homocysteine (Hcy) remethylation: folate provides 5-methyltetrahydrofolate (5-MTHF) as the methyl donor, and B12 is the transferring cofactor for methionine synthase. By splicing a methyl group onto the cysteine backbone, homocysteine is converted to methionine, which is essential for initiating protein synthesis, regulating gene expression via methylation, and maintaining cellular antioxidant defenses.

Elevated homocysteine is a marker for impaired methionine synthesis. Elevated plasma Hcy (>12–15 μmol/L) exerts multiple toxic effects on the retinal vasculature [44,45,46,47]:

- Direct endothelial cytotoxicity via reactive oxygen species (ROS) generation and thiol-disulfide redox stress, disrupting the inner blood–retinal barrier (BRB) [44].
- Promotion of a prothrombotic state through impaired nitric oxide (NO) bioavailability, platelet hyperactivation, and upregulation of coagulation factors, predisposing to retinal vascular occlusions [46].
- Activation of NMDA (N-methyl D-aspartate) receptors on retinal ganglion cells and Müller glia, inducing excitotoxic calcium influx and apoptosis [46].
- Disruption of retinal pigment epithelium (RPE) structure and function, inducing AMD (age-related macular degeneration)-like changes including basal laminar deposits and complement dysregulation [44].

### 3.2. Mitochondrial Dysfunction and Energy Failure

Folate is also required for mitochondrial one-carbon metabolism. Specifically, mitochondrial folate-dependent serine hydroxymethyltransferase (SHMT2) generates formate from serine, which enters the cytosolic folate cycle to support purine synthesis and methylation. In folate deficiency, formate accumulates in a toxic form, directly inhibiting cytochrome c oxidase (complex IV) of the electron transport chain (ETC) [48]. B12 deficiency independently disrupts propionate catabolism (via methylmalonyl-CoA mutase), leading to faulty myelination of optic nerve ganglion cell axons, odd-chain fatty acid accumulation and further ETC impairment [2].

Riboflavin (B2) deficiency exacerbates these effects by impairing MTHFR activity (which requires FAD) and ETC complexes I and II, while pyridoxine (B6) deficiency compromises the transsulfuration pathway [3]. Maculopapular bundle (MP bundle) RGCs, which have the highest mitochondrial density and axonal metabolic demand of all retinal neurons, are selectively vulnerable to this energy failure, explaining the characteristic bilateral centrocecal scotoma of nutritional optic neuropathy [2,9,49].

### 3.3. eNOS Uncoupling and Vasoconstriction (DHFR-Related)

The enzyme dihydrofolate reductase (DHFR) is required both to activate dietary folic acid (dihydrofolate → THF) and to recycle the endothelial nitric oxide synthase (eNOS) cofactor tetrahydrobiopterin (BH2 → BH4). The common DHFR 19 bp deletion polymorphism impairs BH4 recycling, causing eNOS uncoupling: instead of generating vasodilatory NO, uncoupled eNOS produces the superoxide anion (O_2_^−^). Superoxide reacts with residual NO to form peroxynitrite (ONOO^−^), causing oxidative endothelial injury, simultaneously depleting NO bioavailability [19]. The net effect is vasoconstriction, increased retinal arteriolar tone, and microvascular ischemia. This is compounded by high folic acid intake. Folic acid supplementation greater than 400 mcg/day is poorly converted, causing unmetabolized folic acid (UMFA) to accumulate. This competitively inhibits DHFR and further reduces BH4 recycling efficiency [19]. If common reduced DHFR variants are present, folic acid intakes of as little as 200 mcg/day cause UMFA accumulation.

### 3.4. FRα Autoantibody-Mediated Transport Blockade

The folate receptor alpha (FRα, encoded by FOLR1) is expressed at high levels in the blood–brain barrier (BBB), choroid plexus, and placental trophoblasts. Blocking and binding FRα autoantibodies (FRAAs) impair receptor-mediated folate transport across the blood–brain and blood–retina barriers and across the placenta. In the brain, FRAA leads to a state of CNS folate deficiency clinically named CFD, a state of inadequate intracellular folate despite normal serum folate levels [17,50]. The retina is within the CNS. In the retina, FRα is also expressed by RPE, RGCs, bipolar cells, Müller glia, and photoreceptors, as demonstrated by recent immunohistochemical localization studies [51]. Concomitantly, the reduced folate carrier (RFC1/SLC19A1) is present in retinal microvascular endothelial cells and regulates BRB folate transport [31]. FRAA-mediated impairment of retinal folate delivery leads to progressive chorioretinal atrophy, RPE degeneration, and optic neuropathy in untreated cases [50].

## 4. Retinal Manifestations by Etiology

### 4.1. Nutritional Folate Deficiency

Dietary or malabsorptive folate deficiency produces a syndrome of nutritional optic neuropathy [1,2,9,52] characterized by:

- Bilateral, symmetric, progressive central and paracentral visual loss with centrocecal scotomas on visual field testing [9,52].
- Dyschromatopsia (especially red-green) and reduced contrast sensitivity, reflecting selective Maculopapular bundle dysfunction [49].
- Temporal optic disc pallor on fundoscopy, with RNFL thinning demonstrable on SD-OCT, particularly in the temporal quadrant [53,54].
- Occasional peripapillary hemorrhages in the context of concurrent macrocytic anemia [2].
- Increased risk of retinal vein and artery occlusions attributable to HHcy-driven prothrombosis [46,55].

Primate studies provide compelling mechanistic and reversibility data: rhesus monkeys maintained on folate-deficient diets develop histopathologically confirmed optic nerve degeneration with maculopapular bundle selectivity, contrast sensitivity loss on behavioral testing, and RNFL thinning on OCT, all of which are reversible with folate repletion [49]. A 2026 mouse study demonstrated that maternal folate-deficient diets cause congenital retinal and optic nerve anomalies in offspring, with OCT-confirmed RNFL and total retinal thickness reductions, worsened by genetic context (SHMT1 knockout) [48].

### 4.2. Components

FRAA-mediated CFD produces a distinct and severe retinal phenotype beyond that of simple folate deficiency, attributable to the combined loss of RPE-mediated ocular folate transport and direct FRα dysfunction within retinal neurons [17,50,51]:

- Chorioretinal atrophy: Pediatric CFD patients with untreated FRAA syndrome develop massive, progressive chorioretinal degeneration affecting both the RPE and the choroidal vasculature [50]. The degree of atrophy correlates with duration of untreated disease.
- Optic atrophy with diffuse RNFL loss and GCC thinning [50].
- Progressive central visual loss that may stabilize or partially reverse with folinic acid (leucovorin) therapy, which bypasses FRα-mediated transport [17].

The anatomic basis for this retinal vulnerability was established by Flood et al. [51], who demonstrated FRα and RFC1 protein expression across multiple retinal cell types by immunohistochemistry, and by Gurler et al. [31], who confirmed RFC1 expression specifically in retinal microvascular endothelial cells and its role in regulating BRB integrity. These findings place CFD-related retinal disease in a mechanistically coherent framework of impaired intraocular folate transport.

### 4.3. MTHFR Polymorphisms (C677T and A1298C)

The MTHFR C677T variant (homozygous TT: ~10% of most populations) reduces enzyme activity by approximately 70%, substantially impairing 5-MTHF production and elevating Hcy [18,55]. Heterozygous mutations are common, at approximately 40% of the population, and result in an estimated 35% reduction in enzyme activity [56,57]. Retinal consequences include:

- Retinal vein and artery occlusions (RVO/RAO), papillophlebitis, and intraretinal hemorrhages, mediated by HHcy-driven thrombophilia [18,55].
- Diabetic retinopathy (DR) progression: MTHFR C677T/A1298C carriers with diabetes show accelerated microvascular pathology [16,18].
- Microvascular structural changes in mouse models: The Mthfr677C>T knock-in mouse demonstrates age- and sex-dependent reductions in retinal vascular density, increased arteriovenous crossings, and vessel tortuosity on high-resolution imaging, directly mirroring human vascular risk [55].
- Reduced retinal tissue perfusion on OCTA in mild DR patients with MTHFR polymorphisms, with genotype-specific improvements following L-methylfolate supplementation [16,18].

### 4.4. DHFR Polymorphisms

The DHFR 19 bp deletion polymorphism impairs both folic acid activation and BH4 recycling. Unlike MTHFR variants, DHFR dysfunction is particularly problematic in populations with high synthetic folic acid intake (e.g., mandatory fortification programs), where UMFA accumulation competitively inhibits residual DHFR activity [19]. The resulting eNOS uncoupling (Section 3.3) contributes to:

- Retinal arteriolar vasoconstriction and reduced capillary perfusion pressure [19].
- Elevated retinal venous pressure (RVP), a measurable correlate of impaired retinal outflow [19].
- Potential moderation of susceptibility to retinoblastoma, with epidemiological evidence of genotype–drug interactions involving DHFR in pediatric cancer risk [19].

### 4.5. Vitamin B12 Deficiency

Vitamin B12 deficiency produces a retinal syndrome indistinguishable from that of folate deficiency by clinical examination alone, underlining the shared mechanistic pathways (HHcy, mitochondrial toxicity, maculopapular bundle vulnerability) [1,2,58]. Quantitative OCT and OCTA characterization has advanced considerably in recent years:

- Peripapillary RNFL thinning: Temporal quadrant thinning is the most consistent finding. In a pediatric cohort, superior and global RNFL were significantly thinner in B12-deficient children versus controls (*p* = 0.007 and *p* = 0.01, respectively), with global and superior RNFL directly correlating with serum B12 levels (r = 0.296 and r = 0.369, respectively) [59]. Adult case series document that there is a mean RNFL reduction of 10–20% in symptomatic patients [54,58].
- Macular GCC/GCL (ganglion cell complex/ganglion cell layer) thinning: Bilateral diffuse GCC loss on macular OCT often accompanies RNFL changes and correlates with centrocecal scotoma location on perimetry [58].
- Reduced peripapillary and macular vessel density on OCTA: Pellegrini et al. provided the first detailed OCTA characterization of B12 deficiency optic neuropathy, demonstrating decreased radial peripapillary capillary (RPC) vessel density at the optic nerve head and maculopapular bundle, with foveal avascular zone (FAZ) enlargement [33].
- Koca et al. [34] confirmed OCTA-detectable RPC vessel density reductions in a B12 deficiency anemia cohort (*n* = 24), even in patients without overt optic neuropathy, suggesting OCTA may detect preclinical microvascular changes.
- Reversibility: OCT/OCTA changes, particularly in the earlier stages, show partial or complete improvement with B12 repletion, reinforcing the importance of early biochemical diagnosis [33,34,58,59].
- Methyl trap (or folate trap): Vitamin B12 deficiency stops conversion of MTHF to THF, elevating homocysteine due to the unavailability of folate [29].

## 5. Multimodal Diagnostic Assessment

Given the overlapping etiologies and the need to detect changes before irreversible axonal loss occurs, a systematic multimodal testing strategy is recommended. Table 1 summarizes the recommended assessment battery.

Taken together, these findings indicate that vitamin deficiencies, particularly folate and B12, are associated with RNFL loss on OCT across a range of etiologies. Nutritional optic neuropathy classically presents with bilateral, symmetrical, progressive visual impairment, dyschromatopsia, and centrocecal scotoma, with disc pallor, disc atrophy, demyelination, loss of retinal ganglion cells, and RNFL loss [2]. This pattern recurs across distinct causes: in diabetic retinopathy, homocysteine elevation, even with normal folate and B12 levels, correlates with RNFL loss [60]; combined iron, folate, and B12 deficiency produces generalized RNFL thinning [61]; and isolated nutritional folate deficiency likewise produces generalized RNFL thinning [53]. The inborn errors of cobalamin metabolism mark the severe end of this spectrum: cobalamin C (cblC) deficiency produces extreme macular thinning with near-complete loss of the outer nuclear layer despite preservation of retinal lamination [62], and early-onset cblC produces a rapidly progressing maculopathy with severe ganglion cell and outer nuclear layer loss, with inner retinal thickening consistent with a remodeling response to photoreceptor and ganglion cell degeneration [63]. We have additionally observed loss of contrast sensitivity, loss of central acuity, and generalized retinal thinning, particularly of the GCL, in patients with riboflavin deficiency (C.B., unpublished observations). Population-based cohort data corroborate these findings: in the Leipzig LIFE-Adult cohort, serum vitamin B12 and folate levels each showed a significant, independent inverse association with outer nuclear layer thickness [64], and in the 646-participant Alienor cohort, higher circulating folate, vitamin D, and vitamin E levels predicted slower RNFL thinning over 10 years of follow-up, suggesting a neuroprotective effect [65].

As good as it is, OCT may be too blunt an instrument to resolve the finer-grained patterns of retinal injury caused by one-carbon metabolism errors, particularly those triggered by FRAA. We thus propose a multimodal approach that combines standard fundus photography, OCT, OCTA, perimetry, and microperimetry with serological and genetic biomarkers to reveal the more subtle patterns. We expect such non-invasive screening may allow nutritional correction before CFD, autism-associated nutritional risk, or nutritional deficiency injury to the retina and optic nerve becomes established.

### 5.1. Workflow Integration

A recommended clinical workflow begins with biochemical and genetic testing at baseline, conducted concurrently with multimodal retinal imaging (OCT/OCTA, wide-field fundus photography, and FAF). Functional testing (visual acuity, color vision, contrast sensitivity, Humphrey fields, and microperimetry) completes the initial evaluation. Electrophysiology (VEP, pERG) is added when subclinical dysfunction is suspected despite intact visual acuity or when other findings are equivocal [2]. Both kinds of perimetry may find abnormalities earlier than clinically visible, early vascular perfusion.

Following metabolic correction (B12 injection, L-methylfolate or folinic acid supplementation for FRAA cases), serial monitoring with OCT and OCTA at 2–6-month intervals enables objective quantification of structural and perfusion recovery [33,34]. Functional testing is repeated at 3-6-12 months. Absence of improvement after adequate metabolic correction should prompt re-evaluation of the diagnosis, including alternative causes of optic neuropathy.

## 6. L-Methylfolate Supplementation: Mechanisms and Retinal Evidence

L-methylfolate (5-methyltetrahydrofolate; 5-MTHF) is the dominant dietary folate. It is the bioactive, blood–brain barrier-penetrant form of folate that directly enters the methylation cycle without requiring MTHFR or DHFR enzymatic activation. This property confers significant advantages over synthetic folic acid in individuals with MTHFR or DHFR polymorphisms, allowing efficient methionine synthesis (thus lowering homocysteine), restored methylation capacity, and eNOS re-coupling via BH4 salvage [16,18,19].

Homocysteine elevation is a biomarker for one-carbon metabolism impairment. Whether it is directly vasculopathic or a biomarker for other vasculopathic entities is still debated. Studies show that folate, and vitamin B2, B6, and B12 individual supplementations lower homocysteine. It is reasonable that supplementing all of the vitamins key to one-carbon metabolism might be as effective or more effective than monotherapy. Wang and co-workers studied an 18-patient cohort of early diabetic retinopathy patients with common MTHFR mutations by measuring multiple vascular perfusion indices and biomarkers, including homocysteine before and after the use of a commercial medical food (Ocufolin^®^) supplying these vitamins and multiple antioxidants [66,67]. This dataset is unique in relating retinal perfusion metrics with metabolic biomarkers before and after supplementation. It is sufficient to be hypothesis generating, suggesting how genetic folate metabolic impairment affects retinal vasculature. Larger controlled trials should calibrate this and be it can useful in modeling the impact of MTHFR polymorphisms on retinal vasculature and perfusion.

- BCVA improvement: Statistically significant gains in best-corrected visual acuity, most pronounced in compound heterozygotes (C677T/A1298C) and C677T homozygotes [16,18].
- OCTA perfusion improvement: Increased vessel density and flow indices; retinal arteriolar blood flow velocity increases as measured by Retinal Function Imager (RFI), with the greatest perfusion gains in the highest-risk genotypes [18].
- Homocysteine reduction: Plasma Hcy reductions of 20–40% (from approximately 14–16 μmol/L to 9–12 μmol/L; *p* < 0.001), more sustained than with synthetic folic acid in polymorphic individuals [16,18].
- Retinal venous pressure (RVP) reduction: In a glaucoma/ocular vascular disease cohort with elevated baseline Hcy (>12 μmol/L), mean RVP dropped by 8.8 mm Hg after 3 months (*p* < 0.001) [18].
- Total retinal blood flow: A positive trend after 4 months and significant improvement in 6 months in diabetic patients when using 5-MTHF supplement supplied from Ocufolin [16,67].

### 6.1. Retinal Vein Occlusion and AMD Applications

In a small case series (*n* = 5 eyes) of retinal vein occlusion patients treated adjunctively with Ocufolin, an approximately 44% reduction in average RNFL thickness and 30% reduction in central macular thickness were observed within 6 months, accompanied by better-than-anticipated BCVA recovery and no progression to ischemic RVO [16]. In neovascular AMD, adjunctive L-methylfolate plus methylcobalamin (B12) with anti-VEGF (vascular endothelial growth factor) therapy further reduced RVP and Hcy while improving macular morphological outcomes and extending treatment intervals [16]. We note that this was an observational study, with data collection beginning after the vein occlusions were detected, so there were no patient specific baseline normals, and the reductions reported were based on data for each patient.

### 6.2. Clinical Considerations

L-methylfolate is generally well tolerated, does not mask B12 deficiency (a concern with synthetic folic acid), and minimizes UMFA accumulation [19]. Typical therapeutic doses range from 400 to 900 μg/day in ocular formulas, often combined with active B2, B6, and B12 cofactors [5,12]. Benefits are most pronounced when baseline Hcy is elevated (>12 μmol/L) or MTHFR/DHFR polymorphisms are present. Individualized dosing guided by laboratory and genetic results is recommended; specialist consultation is advised for complex cases.

## 7. A Machine Learning Framework for Retinal Detection of Cerebral Folate Deficiency

### 7.1. Current State of AI in Retinal Imaging for Nutritional and Metabolic Disease

Artificial intelligence and deep learning have transformed retinal disease screening over the past decade, with regulatory-approved systems for diabetic retinopathy, AMD, and glaucoma. More recently, DL models have demonstrated the capacity to detect systemic metabolic states from retinal images alone, representing the retina as a “window” to systemic health.

Critically relevant foundational work includes:

- Anemia detection from fundus images: Mitani et al. [35] demonstrated that DL models trained on retinal fundus photographs could detect anemia and estimate hemoglobin concentration with high accuracy (area under the curve (AUC) > 0.88), identifying the optic disc and peripapillary region as the most discriminative areas. Because macrocytic anemia from B12 and folate deficiency produces characteristic vascular and disc changes—pallor, vascular tortuosity, altered vessel caliber—this approach has direct relevance to nutritional deficiency screening [68,69,70].
- Multi-disease retinal screening: Dong et al. [36] demonstrated a DL system capable of simultaneously screening for more than 10 retinal and systemic conditions, including categories encompassing nutritional and toxic optic neuropathies, with high sensitivity and specificity.
- Neuro-ophthalmic disorder classification: Szanto et al. [71] reported a DL system achieving 93–96% accuracy in differentiating optic disc swelling etiologies from fundus photographs, a framework directly applicable to optic neuropathy triage including nutritional causes.
- LLM-assisted differential diagnosis: Shukla et al. [72] evaluated large language models for AI-assisted differential diagnosis of retinal and neuro-ophthalmic disorders, including nutritional optic neuropathy, demonstrating growing AI capacity in this clinical domain.
- MTHFR-specific perfusion imaging: Jiang et al. [18] and Liu et al. [16] quantified OCTA perfusion metrics in MTHFR-variant DR patients, generating quantitative feature sets (vessel density maps, arteriolar flow velocity) that constitute ideal ML training inputs for genotype-specific phenotyping.
- Vascular cognitive impairment detection using OCTA measures of retinal vessel density: Zhou et al. [73] evaluated OCTA from cognitively normal and impaired subjects without vision disorders and found a correlation of decreasing retinal vessel density with increasing cognitive impairment.
- Selective ASD determination in children aged 2–6: Zee et al. [43] trained an ML system on color fundus images of children with neurodevelopmental disorders and matched controls (2000+ total subjects). Their ML system successfully isolated three developmental disabilities from control and from each other, using multiple retinal parameters. While they have no FRAA data for these subjects, the ability to identify ASD selectively from global developmental delay or from the combination of these two conditions bodes well for our proposal here to extract CFD evidence from retinal images of ASD-relevant subjects.

### 7.2. Proposed ML Architecture for CFD Retinal Screening

We propose a multimodal, hierarchical ML framework for CFD detection, structured around four components:

#### 7.2.1. Input Data Modalities

The model would integrate the following input streams:

- Structural OCT: Peripapillary RNFL thickness maps (all quadrants), macular GCC/GCL thickness maps, optic disc segmentation parameters [53,58,59].
- OCTA perfusion: Optic nerve head and superficial/deep macular vessel density maps, FAZ area and perimeter, radial peripapillary capillary (RPC) density, choriocapillaris flow deficit maps [33,34].
- Color fundus photography: Disc pallor index, vascular caliber ratios, hemorrhage detection [35,36].
- Functional metrics (optional): BCVA, Humphrey mean deviation and pattern standard deviation, microperimetry mean sensitivity, contrast sensitivity [49].
- Biochemical and genetic metadata (optional): Serum/RBC folate, B12, B6, riboflavin, vitamin D, plasma Hcy, MMA, MTHFR/DHFR genotype, FRAA result, as auxiliary inputs for outcome-guided training and risk stratification [17,18,19].

We note that OCTA data may be the most information-rich scan, as seen in Table 2, but it does not image larger-scale structural conformations as well as traditional OCT and traditional imaging. Also, OCTA is an expensive technology that most practitioners do not have common access to. Finding markers with OCT and traditional images that are validated with OCTA data would be very useful in identifying at risk patients. We thus propose a multimodal data collection of laboratory, genetic, traditional fundus imaging, visual field, OCT structural scanning, and OCTA vascular imaging and perfusion scanning. Each provides unique information. We propose that the whole may be more than the sum of the parts, allowing patterns to be identified that may not be apparent with any single image format.

#### 7.2.2. Model Architecture

A convolutional neural network (CNN) or Vision Transformer (ViT) backbone would process imaging inputs, with separate branches for OCT structural maps and OCTA perfusion maps fused via a multimodal attention mechanism. Biochemical and genetic metadata would be incorporated as tabular auxiliary inputs through a multi-layer perceptron branch, with late fusion to the image-based embeddings. Transfer learning from pre-trained models for diabetic retinopathy, optic neuropathy, and anemia detection [35,36,37] would mitigate the expected data scarcity of labeled CFD-specific datasets.

#### 7.2.3. Output and Clinical Integration

The model would output a continuous CFD risk probability score and a categorical classification (low/intermediate/high risk), with spatial attention maps (e.g., Grad-CAM) highlighting retinal regions driving the classification. Integration with electronic health records and prenatal care platforms could enable automated referral triggers when retinal screening flags elevated CFD risk. A cloud-based implementation would allow deployment in resource-limited settings via teleophthalmology infrastructure.

#### 7.2.4. Training and Validation Strategy

The primary training target would be FRAA positivity as a surrogate label for CFD [17,38], with secondary labels including MTHFR genotype, plasma Hcy tertile, and OCT/OCTA metric abnormality thresholds. Validation would require multi-site prospective cohorts with paired retinal imaging and biomarker data, with independent test sets stratified by genotype and clinical syndrome. Performance benchmarks should include sensitivity ≥ 90% for FRAA positivity and AUC ≥ 0.85 for CFD risk stratification. Regulatory-grade validation would follow prospective enrollment in a dedicated clinical trial. Table 3 compiles a listing of the potential measurements and how strong the evidence is, or is expected to be, for their use in diagnosing CFD.

#### 7.2.5. Extended Applications

It has not escaped our attention that this method of detecting alterations in one-carbon metabolism could be a means to flag vitamin-deficient retinopathies that can facilitate an early diagnosis of other conditions. Disorders including glaucoma, AMD, diabetic neuropathies, cognitive decline, and several other conditions, including some autoimmune disorders, likely have related manifestations that are visible in retinal imagery. Using a non-invasive retinal scan could be a means to assess for vitamin deficiencies that could be addressed to restore metabolic parameters in the 1C cycle and minimize or possibly reverse a disease state.

## 8. Application to Prenatal Screening and Autism Spectrum Disorder Risk

A compelling translational application of the retinal CFD detection framework, once developed and validated, is prenatal risk stratification for autism spectrum disorder (ASD). Epidemiological and mechanistic evidence supports a folate metabolism–ASD risk association [38,39,74,75]. The application we describe below builds on that association and is not as yet a validated screening tool.

### 8.1. Folate Metabolism as an ASD Risk Pathway

Pooled prevalence of FRAAs in ASD cohorts ranges from 38% to 70% across multiple studies, substantially exceeding the estimated 2–3% prevalence in the general population [38]. Maternal FRAAs impair placental and choroid plexus folate transport during gestation, potentially depriving the developing fetal brain of adequate folate for DNA methylation, myelination, and neurotransmitter synthesis [17,38,76]. Epidemiological data from Giorlandino et al. [74] demonstrate that FRAA-positive pregnancies are associated with increased fetal nuchal translucency and higher rates of pregnancy complications including early miscarriage, neural tube defects, and oligohydramnios. In our Supplemental Materials, Table S2 provides a listing of key research findings from publications examining CFD in an ASD population.

MTHFR C677T is associated with elevated ASD risk, particularly in populations without mandatory folate fortification [39]. Frye et al. [75] have further characterized the interaction between maternal MTHFR genotype, folate intake form (synthetic vs. L-methylfolate), and neurodevelopmental outcomes, supporting genotype-guided prenatal supplementation strategies.

### 8.2. Retinal Imaging as a Prenatal and Neonatal Biomarker

FRα expression in the RPE [51] creates a direct mechanistic link between maternal/neonatal retinal pathology and systemic/cerebral folate transport capacity. Maternal OCT and OCTA performed during pregnancy is safe (non-ionizing, non-contact) and can detect RNFL thinning, GCC loss, vessel density reduction, or FAZ changes as retinal indicators of systemic FRα dysfunction and HHcy [33,34]. Neonatal/infant OCT performed post-partum may reveal early chorioretinal or maculopapular changes as proxies for in utero CFD exposure [50].

### 8.3. An Integrated Prenatal CFD Screening Protocol

We propose that a practical prenatal screening protocol integrate:

1. First-trimester laboratory screening: FRAA (blocking and binding FRAAs), MTHFR/DHFR genotyping, plasma Hcy, serum/RBC folate, and full B-vitamin and vitamin D panel [17,38,74].
2. First-trimester retinal imaging: SD-OCT and OCTA in FRAA-positive or genetically high-risk mothers to assess baseline retinal structure and perfusion [33,34].
3. Targeted metabolic intervention: Periconceptional and prenatal supplementation with L-methylfolate (or folinic acid to bypass FRα blockade in FRAA-positive cases); avoidance of high-dose synthetic folic acid in DHFR variant carriers [19,75].
4. Serial retinal monitoring: OCT/OCTA at each trimester in high-risk patients to track retinal response to supplementation and confirm metabolic improvement [16,18].
5. Neonatal retinal screening: Infant OCT (portable handheld devices) in offspring of FRAA-positive mothers to identify early chorioretinal changes and prompt folinic acid initiation [50]. A recent OCT study of very preterm (<32 weeks gestational age) infants found that thicker retinal nerve fiber layers were correlated with higher Bayley Scales of Infant and Toddler Development, with a lower risk of autism at 2 years, thus documenting that even the youngest infants may have measurable retinal differences that can be detected with OCT [77].

Meta-analyses of periconceptional folate supplementation consistently demonstrate reductions in ASD incidence [39,75]; genotype-guided use of L-methylfolate or folinic acid is expected to further optimize outcomes in the highest-risk genetic subgroups. An ML model trained on maternal and neonatal retinal images paired with FRAA outcomes and offspring neurodevelopmental diagnoses would constitute the definitive validation dataset for this approach.

## 9. Research Gaps and Future Directions

We identified several critical gaps that represent high-priority targets for future research:

### 9.1. Labeled Retinal Imaging Datasets

No large-scale, publicly available OCT or OCTA dataset currently includes images labeled specifically for folate deficiency genotype (MTHFR, DHFR), FRAA status, or CFD diagnosis. The construction of such a dataset—ideally multi-site, prospective, and including paired biochemical, genetic, retinal imaging, and functional data—is the single most important enabling step for ML model development in this domain [35,36].

### 9.2. Standardization of OCT/OCTA Metrics

Quantitative RNFL and vessel density thresholds for B12 and folate deficiency optic neuropathy remain unstandardized across imaging platforms (Heidelberg Spectralis, Zeiss Cirrus, Optovue Avanti). Multicenter normative data and cross-platform harmonization are needed for ML generalizability [33,34].

### 9.3. Longitudinal Recovery Studies

Prospective studies that track OCT/OCTA metrics, functional outcomes, and biochemical parameters through a full course of metabolic correction (B12 injection, L-methylfolate, or folinic acid) with predefined follow-up intervals are needed to define the kinetics of retinal recovery and establish prognostic imaging biomarkers [16,18,33].

### 9.4. Pediatric and Neonatal Normative Data

Handheld OCT normative datasets for infants and young children are limited. The development of age-stratified pediatric retinal imaging norms is essential for validating neonatal CFD screening protocols [50,59].

### 9.5. Randomized Controlled Trials of L-Methylfolate

Current evidence for retinal perfusion benefits of L-methylfolate derives predominantly from small pilot studies and observational series [16,18]. Adequately powered RCTs (randomized controlled trials) with prespecified OCTA endpoints, stratified by MTHFR/DHFR genotype and baseline Hcy, are needed to establish definitive treatment guidelines.

## 10. Conclusions

Folate and vitamin B12 metabolic dysfunction, encompassing nutritional deficiencies, FRα autoantibody syndromes, and MTHFR/DHFR polymorphisms, produces a spectrum of retinal changes that are quantifiable, reproducible, and frequently reversible with early metabolic correction [2,9,33,34,49,52,53,59]. The convergent pathophysiological mechanisms (hyperhomocysteinemia, mitochondrial dysfunction, eNOS uncoupling, and FRα transport blockade) generate measurable retinal phenotypes across structural (RNFL/GCC thinning, chorioretinal atrophy), microvascular (vessel density reduction, FAZ enlargement, capillary dropout), and functional (centrocecal scotoma, dyschromatopsia, reduced contrast sensitivity) domains [16,44,45,46,50].

Modern multimodal retinal imaging, particularly SD/SS-OCT for structural analysis and OCTA for non-invasive perfusion mapping, provides the quantitative measurement infrastructure needed to detect these changes in research and clinical settings [33,34,58,59]. Concurrent biochemical and genetic testing enables etiological precision [17,18,19].

**Figure 2:**
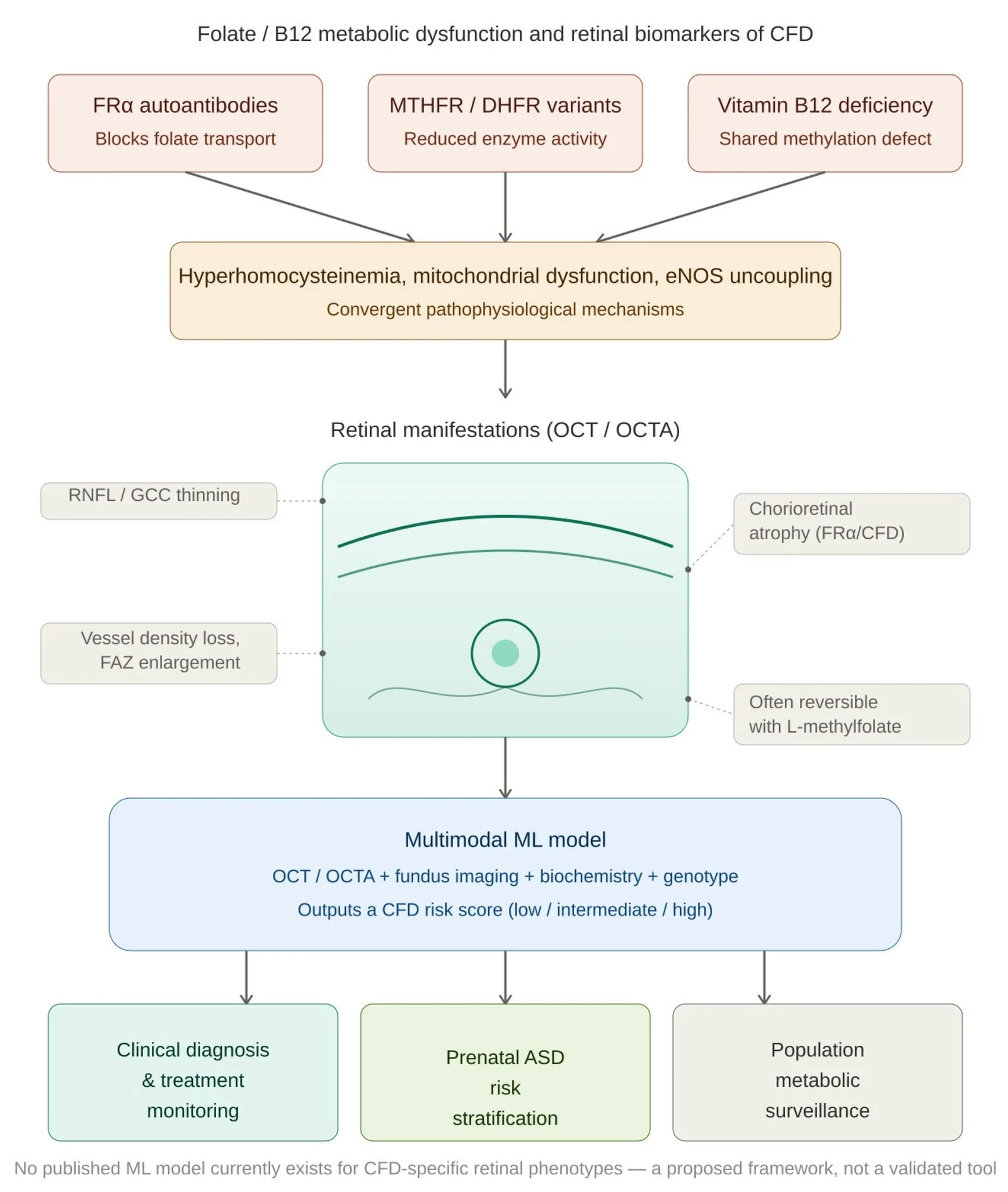
Schematic representation of the rationale for a machine learning retinal detection method of cerebral folate deficiency in ASD. Image depicts the inputs that may alter retinal folate distribution, resulting in dysfunctions that are manifested in retina and detectable with a machine learning model using retinal images (fundus, OCT, OCTA) to provide a folate deficiency risk score that, if verified, could lead to diagnosis and treatment outcomes. Colors follow a gradient, from biological cause (pink) to retinal readout (green), to computational model (blue) to clinical output (mixed).

The proposed ML framework, summarized in Figure 2, represents a logical extension of established deep learning architectures for retinal anemia detection and optic neuropathy classification [35,36,37], but it remains conceptual: no labeled CFD-specific retinal dataset yet exists to train such a model. Its potential clinical applications could span individual diagnosis, treatment response monitoring, prenatal ASD risk stratification [38,39,74,75], and population metabolic surveillance. The critical prerequisite is the prospective construction of adequately powered, multi-site retinal imaging datasets with linked biochemical and genetic annotation.

The retina’s accessibility, metabolic sensitivity, and imaging precision make it a uniquely powerful portal for detecting cerebral and systemic metabolic dysfunction, a potential paradigm shift in non-invasive biomarker discovery for folate-related neurological disease.

## Figures and Tables

**Table 1:** Recommended multimodal assessment battery for suspected B-vitamin metabolic optic neuropathy.

Domain	Test/Modality	Key Metrics and Clinical Relevance
Biochemistry	Serum and RBC folate; plasma Hcy; methylmalonic acid (MMA)	Establishes folate/B12 status; elevated Hcy and MMA indicate functional B12 deficiency; RBC folate reflects tissue stores
Biochemistry	Vitamin panel (B12, B6, B2)	Identifies overlapping deficiencies amplifying retinal/vascular risk
Genetics	MTHFR C677T/A1298C genotyping; DHFR 19 bp deletion; there are rare variants with limited data	Identifies enzymatic inefficiency; guides preference for L-methylfolate over synthetic folic acid
Immunology	FRα autoantibody (FRAA assay): blocking and binding	Confirms FRAA-mediated CFD; highest prevalence in ASD and neurological regression populations
Structural OCT	SD/SS-OCT: peripapillary RNFL (4 quadrants); macular GCC/GCL; optic disc morphology	RNFL temporal thinning = maculopapular bundle loss; GCC thinning = early macular RGC involvement; serial monitoring tracks progression/recovery
Perfusion OCTA	OCTA (preferred): optic nerve head and macular vessel density; FAZ area/perimeter; non-perfusion zones	Quantifies capillary-level ischemia; detects HHcy/eNOS uncoupling-related microvascular loss; ideal for L-methylfolate response monitoring
Wide-field imaging	Wide-field fundus photography; fundus autofluorescence (FAF)	Documents disc pallor, hemorrhage, RPE change (chorioretinal atrophy in CFD); FAF highlights RPE metabolic stress
Visual function	Best-corrected visual acuity (BCVA); color vision (Ishihara, Farnsworth-Munsell 100-Hue); contrast sensitivity (Pelli-Robson, Mars)	BCVA: overall visual impact; color: dyschromatopsia of maculopapular bundle; contrast: sensitive early functional marker
Perimetry	Automated visual fields (Humphrey 24-2); microperimetry (MAIA or MP-3)	Centrocecal scotoma mapping; microperimetry provides fundus-correlated sensitivity data for maculopapular bundle region
Electrophysiology	Visual evoked potentials (VEP); pattern ERG (pERG)	VEP: optic nerve conduction; pERG: RGC function; both sensitive for subclinical dysfunction

RBC = red blood cell; Hcy = homocysteine; MMA = methylmalonic acid; RNFL = retinal nerve fiber layer; GCC = ganglion cell complex; GCL = ganglion cell layer; RGC = retinal ganglion cell; FAZ = foveal avascular zone; RPE = retinal pigment epithelium; CFD = cerebral folate deficiency; BCVA = best-corrected visual acuity; VEP = visual evoked potential; ERG = electroretinogram.

**Table 2:** Summary of key OCT and OCTA metrics in folate and B12 metabolic optic neuropathy.

Etiology	Primary OCT Finding	Primary OCTA Finding	Pattern/Selectivity	Key References
Folate deficiency	Temporal RNFL thinning; RNFL loss all quadrants (severe)	Not yet characterized by OCTA specifically	Maculopapular bundle; bilateral symmetric	[49,52,53]
B12 deficiency	Temporal >> superior RNFL thinning; macular GCC/GCL loss	Reduced RPC VD (ONH); macular VD decrease; FAZ enlargement	Maculopapular bundle; bilateral symmetric	[33,34,59,72]
FRα autoantibody/CFD	Diffuse RNFL and GCC thinning; chorioretinal atrophy on OCT-B scan	Choriocapillaris flow deficits; RPE disruption on en face OCT	Diffuse; may be asymmetric; severe in untreated pediatric CFD	[50,51]
MTHFR polymorphism	Reduced RNFL/GCC in polymorphic DR patients; vascular tortuosity in mouse model	Reduced macular and peripapillary VD; arteriolar flow velocity decrease	Genotype-dependent; worse in TT homozygotes; DR-context predominant	[16,18,55]
Hyperhomocysteinemia (all etiologies)	RNFL thinning correlating with Hcy level; GCC loss	Reduced vessel density; FAZ area increase; BRB disruption	Temporal maculopapular bundle; microvascular; correlates with Hcy	[44,45,46]

RNFL = retinal nerve fiber layer; GCC = ganglion cell complex; GCL = ganglion cell layer; RPC = radial peripapillary capillary; VD = vessel density; ONH = optic nerve head; FAZ = foveal avascular zone; RPE = retinal pigment epithelium; CFD = cerebral folate deficiency; DR = diabetic retinopathy; BRB = inner blood–retinal barrier; Hcy = homocysteine. Reference numbers correspond to the Reference list.

**Table 3:** Proposed multimodal ML feature set for CFD retinal screening, highest evidence strength listed at top.

Input Modality	Feature/Metric	Pathophysiological Correlate	Evidence Strength
Biochemical	FRAA result (blocking/binding)	FRAA-mediated CFD confirmation	High (gold standard)
Biochemical	Plasma homocysteine	Severity of metabolic dysfunction	High
OCTA	RPC vessel density (ONH)	Peripapillary capillary dropout	High [33,34]
SD/SS-OCT	Temporal RNFL thickness	Maculopapular bundle axonal loss	High (multiple cohorts)
SD/SS-OCT	Macular GCC/GCL thickness	RGC body loss; early central involvement	High (multiple cohorts)
Genetic	MTHFR C677T/A1298C status	Enzymatic risk stratification	Moderate–High
SD/SS-OCT	Global RNFL thickness	Diffuse optic neuropathy extent	Moderate–High
OCTA	Macular vessel density (SVP/DVP)	HHcy-driven microvascular ischemia	Moderate–High
OCTA	FAZ area and perimeter	Central capillary dropout	Moderate [33]
OCTA	Choriocapillaris flow deficits	RPE/choroid involvement in CFD	Moderate (emerging)
Fundus photo	Disc pallor index	Advanced RGC and axonal loss	Moderate (clinical)
Fundus photo	Vascular caliber ratio	HHcy-driven arteriovenous changes	Moderate

RNFL = retinal nerve fiber layer; GCC = ganglion cell complex; GCL = ganglion cell layer; RGC = retinal ganglion cell; RPC = radial peripapillary capillary; ONH = optic nerve head; SVP = superficial vascular plexus; DVP = deep vascular plexus; FAZ = foveal avascular zone; RPE = retinal pigment epithelium; HHcy = hyperhomocysteinemia; CFD = cerebral folate deficiency; FRAA = folate receptor alpha autoantibody.

## Data Availability

No new data were created or analyzed in this study. Data sharing is not applicable to this article.

## Supplementary Materials

The following supporting information can be downloaded at https://www.mdpi.com/article/10.3390/cells15161453/s1, Table S1: Summary of 32 publications using AI or ML, grouped by disorder and data category, with focus on autism, as well as with retinal detection methods. One report (Lai et al., 2020) used fundus images to identify whether a child was ASD or not, but no studies have used retinal images to identify cerebral folate deficiency or other nutrient deficiencies [43]; Table S2: Cerebral folate deficiency (CFD): key research findings. References [78,79,80,81,82,83,84,85,86,87,88,89,90,91,92,93,94,95,96,97,98,99,100,101,102,103,104,105,106,107,108,109,110,111,112,113,114,115,116,117,118] were cited in the Supplementary Materials.

## Abbreviations

The following abbreviations are used in this manuscript:

5-MTHF	5-methyltetrahydrofolate
AI	artificial intelligence
AMD	age-related macular degeneration
ASD	autism spectrum disorder
AUC	area under the curve
BBB	blood–brain barrier
BCVA	best-corrected visual acuity
BH4 (BH2)	tetrahydrobiopterin (dihydrobiopterin)
BRB	inner blood–retinal barrier
CFD	cerebral folate deficiency
CNN	convolutional neural network
DHFR	dihydrofolate reductase
DL	deep learning
DR	diabetic retinopathy
DVP	deep vascular plexus
eNOS	endothelial nitric oxide synthase
ERG	electroretinogram
ETC	electron transport chain
FA	fluorescein angiography
FAF	fundus autofluorescence
FAZ	foveal avascular zone
FOLR1	gene encoding folate receptor alpha
FRAA(s)	folate receptor alpha autoantibody(ies)
FRα	folate receptor alpha
GCC	ganglion cell complex
GCL	ganglion cell layer
Hcy	homocysteine
HHcy	hyperhomocysteinemia
IOVS	Investigative Ophthalmology & Visual Science
ML	machine learning
MMA	methylmalonic acid
MP bundle	maculopapular bundle
MTHFR	methylenetetrahydrofolate reductase
NMDA	N-methyl-D-aspartate
NO	nitric oxide
OCT	optical coherence tomography
OCTA	optical coherence tomography angiography
ONH	optic nerve head
ONOO^−^	peroxynitrite
O_2_^−^	superoxide anion
pERG	pattern electroretinogram
PMC	PubMed Central
RAO	retinal artery occlusion
RBC	red blood cell
RCT(s)	randomized controlled trial(s)
RFC1/SLC19A1	reduced folate carrier 1 (gene)/ solute carrier family 19 member 1 (gene)
RFI	Retinal Function Imager
RGC(s)	retinal ganglion cell(s)
RNFL	retinal nerve fiber layer
ROS	reactive oxygen species
RPC	radial peripapillary capillary
RPE	retinal pigment epithelium
RVO	retinal vein occlusion
RVP	retinal venous pressure
SD-OCT/SS-OCT	spectral-domain/ swept-source optical coherence tomography
SHMT1/SHMT2	serine hydroxymethyltransferase (isoform 1)/ serine hydroxymethyltransferase (isoform 2)
SVP	superficial vascular plexus
THF	tetrahydrofolate
UMFA	unmetabolized folic acid
VD	vessel density
VEGF	vascular endothelial growth factor
VEP	visual evoked potential
ViT	Vision Transformer
