# Supplementary material for: Retinal Biomarkers of Folate and Vitamin B12 Metabolic Dysfunction: A Framework for Machine Learning-Assisted Detection of Cerebral Folate Deficiency
Source: Cells. 2026 Aug 13;15(16):1453. doi: 10.3390/cells15161453 (PMC13510911; doi:10.3390/cells15161453)
Supplement: Supplementary file 1 [file cells-15-01453-s001.zip › cells-4471538-supplementary.pdf]

Supplemental Files

Table S1. Summary of 32 publications using AI or ML, grouped by disorder and data category, with focus on autism, as well as with retinal detection methods. One report (Lai et al., 2020) used fundus images to identify whether a child was ASD or not, but no studies have used retinal images to identify cerebral folate deficiency or other nutrient deficiencies.

Disorder/ Data Category	Data Type/Input	Key Finding	Source
**Retinal Diseases (autism-related biomarkers)** Medical Imaging	Retinal imaging	Retinal ML differentiates ASD from non-autistic children using retinal fundus images	Lai et al., 2020 [1]
**ASD** Biologics	Urine metabolomics	Urine test for ASD biomarkers shows ~93% accuracy in small-cohort study	Flynn et al, 2026 [2]
	Plasma proteomics	Identifies ASD-associated plasma protein biomarkers from proteomic profiles	Tang et al., 2025 [3]
	Gut microbiome metagenomics	AUC 0.99 identifying ASD-associated microbial taxa; XAI explains top features	Temiz et al., 2025 [4]
	Whole-exome sequencing (WES)	Predicts ASD status from WES in 43,203 individuals (16,809 ASD); promising genetic classifier	Wang et al., 2024 [5]
	Maternal/infant biomarkers + EHR clinical data	Predicts ASD vs. MSDD using maternal/infant data + standardized test items	Briguglio et al., 2023 [6]
	Early biomarkers (immune, metabolic, environmental exposures)	Reviews maternal, paternal, placental, amniotic biomarkers that predict ASD risk	Rossignol & Frye, 2025 [7]
**ASD** Neuroimaging	Resting-state fMRI	Systematic review: DL on rs-fMRI achieves strong ASD classification; ABIDE dataset widely used [Review of automated ASD diagnosis using rs-fMRI + DL	Huda et al, 2024 [8]
	Structural MRI (sMRI)	Review of non-invasive imaging (sMRI) for early ASD detection; highlights AI models	Abdelrahim et al., 2025 [9]
	fMRI + sMRI (multimodal)	Survey: ML on functional and structural MRI achieves high ASD/ADHD classification; DL outperforms traditional ML	Mertz, 2024 [10]
	EEG	EEG-based ML classifiers distinguish ASD from controls with high accuracy	Kim et al., 2025 [11]
**ASD** Eye Tracking/Gaze	Eye-tracking scanpaths and fixation data	Gaze patterns reliably distinguish ASD; multiple studies >85% accuracy; scanpath images clustered with deep autoencoder	Elbattah et al., 2024 [12]
**ASD** Behavioral/Video	Home videos of children (semi-structured observation)	Questionnaire + video ML algorithm achieves high ASD detection outside clinical settings; mobile screening feasible	Tariq et al., 2018 [13]
	Brief clinical observation video (BOSCA toddlers)	ML on brief socio-communicative behavior observations in <3-year-olds screens for ASD and predicts cognitive developmental trajectory	Baran & Cetin, 2025 [14]

	Semi-structured naturalistic interaction video / reciprocity coding	ML classification of ASD based on reciprocity in naturalistic social interactions is feasible	Pandya et al., 2023 [15]
**ASD** Questionnaires/Clinical Tests	Parent-reported questionnaires (background & medical info, SPARK database)	Robust ML prediction of ASD from minimal background/medical information; best for higher-symptom individuals	Rajagopalan et al., 2024 [16]
	ADOS-2 structured observation + ADI-R parental interview	5-feature subset from ADOS+ADI-R differentiates ASD from ADHD/no-diagnosis with ~92% item reduction	Wolff et al., 2022 [17]
	EHR clinical notes (NLP)	Transparent deep learning on EHR clinical notes identifies ASD with interpretable rationale	Leroy et al., 2024 [18]
	EHR data (child-mother linked records)	Linking maternal EHR to child outcomes improves ASD prediction; helps detect girls diagnosed late	Li et al., 2024 [19]
	Sensory processing surveys + behavioral questionnaires	ML links atypical sensory processing to behavioral problems (aggression, anxiety, attention) in ASD	Mohammadi et al., 2025 [20]
**ASD** Review/Multimodal	Multiple modalities (neuroimaging, genomics, questionnaires, video, eye-tracking)	Comprehensive review of ML in ASD screening, diagnosis, stratification, and treatment; highlights multimodal AI integration and digital phenotyping	Reilly et al., 2025 [21]
	MRI, facial images, questionnaires, EEG, eye tracking	Survey of ASD detection via diverse technologies; DL on fMRI and video outperforms earlier methods	Rajagopalan & Ghosh, 2025 [22]
	All AI approaches (bibliometric)	1,357 papers; rapid growth post-2017; ML on MRI/fMRI and behavioral data dominate; top researchers in US, China, India	Xu et al. 2025 [23]
	AI + genetic/neuroimaging biomarkers	Reviews AI for early ASD identification across imaging, genetics, and behavioral biomarkers	Aldakhil & Alasim, 2025 [24]
	Explainable AI across data types (XAI)	XAI improves transparency of ASD classifiers; SHAP/LIME most common; pediatric populations predominate	Agrawal et al., 2025 [25]
	ML use of computer vision on tablet to screen toddlers for autism	Mobile app uses computer vision and ML to screen for ASD in toddlers.	Babu et al., 2024 [26]
	AI approaches (systematic review - AI in ASD + gene contribution)	AI-based triage and priority frameworks for ASD diagnosis; genes contribute significantly to ML models	Wankhede et al., 2024 [27]
**ASD/ADHD (differential)** Questionnaires/Clinical Tests	Social Responsiveness Scale (SRS) - behavioral ratings	ML on SRS items distinguishes ASD from ADHD; foundational ML-ASD study.	Duda et al., 2016 [28]
	Co-occurring ASD+ADHD survey data	Differential classification of individual ASD vs. ADHD diagnoses from survey data is challenging but feasible with ML	Rakotomanana & Rouhafzay, 2025 [29]
**ASD/ADHD (differential)** Neuroimaging	fMRI + sMRI	ML on fMRI/sMRI can classify ASD and ADHD; deep learning outperforms traditional ML	Eslami et al., 2021 [30]
**ASD/Schizophrenia/Social Anxiety (differential)**	Self-report questionnaires (SPQ-BR, Autism Quotient)	ML identifies overlapping and distinctive traits of ASD vs. schizophrenia; guidance for reducing misdiagnosis	Alsharif et al., 2025 [31]

Questionnaires/Clinical Tests			
**ASD/Early Psychosis/Social Anxiety (differential)** Questionnaires/Clinical Tests	Clinical assessments (cognitive, social, psychiatric)	ML differentiates ASD, early psychosis, and social anxiety disorder from overlapping social difficulty profiles	Demetriou et al., 2020 [32]

Table S2: Cerebral Folate Deficiency (CFD): Key Research Findings

Major Findings	Authors/Year	Sample Size	Source	Publication Type
First description of CFD as a distinct neurometabolic condition: low 5-methyltetrahydrofolate in cerebrospinal fluid causing psychomotor retardation, spastic paraplegia, cerebellar ataxia, and dyskinesia — responsive to folinic acid substitution.	Ramaekers et al., 2002	5 children (case series)	[33]	Mechanistic Study
Early foundational description characterizing CFD as low CSF 5-MTHF with normal serum folate, establishing the diagnostic and clinical framework for the syndrome.	Ramaekers & Blau, 2004	N/A (narrative review)	[34]	Mechanistic Study
A milk-free diet significantly downregulated folate receptor autoantibody (FRAA) titers in children with CFD syndrome, implicating a dietary trigger (cow's-milk protein) for the autoimmune blockade of folate transport.	Ramaekers et al., 2008	24 children (12 milk-free + 12 control)	[35]	Mechanistic Study
Measured serum FRA concentrations in children with ASD and found a high prevalence (75.3%) of FRAs, with FRA concentration correlating significantly with reduced CSF 5-MTHF in a subset who underwent lumbar puncture.	Frye et al., 2013	93 children (16 with CSF correlation)	[36]	Mechanistic Study
Reanalysis distinguishing blocking vs. binding FRAA subtypes, proposing FRAA-positive subtyping of ASD as a basis for more precise, personalized treatment.	Frye et al., 2016	93 children (same cohort as Frye 2013)	[37]	Mechanistic Study
Narrative review of folate metabolism abnormalities in autism, evaluating FRAA, MTHFR variants, and related markers as potential clinical biomarkers.	Frye et al., 2017	N/A (narrative review)	[38]	Mechanistic Study

Case report of chorioretinal atrophy in a pediatric CFD patient, describing this as a preventable retinal manifestation of untreated FRAA-mediated folate transport blockade.	Kakkassery et al., 2021	1 patient (case report)	[39]	Mechanistic Study
Reduced folate carrier (RFC1) shown to be expressed in retinal microvessels and essential for inner blood–retina barrier integrity — mechanistic basis for retina as a proxy tissue for cerebral folate transport.	Gürler et al., 2023	Adult mice (group sizes not uniformly reported)	[40]	Mechanistic Study
Among pregnancies with isolated increased fetal nuchal translucency and negative genetic testing, all 4 FRAA-positive mothers had offspring later diagnosed with ASD, versus 1 of 7 FRAA-negative mothers — proposing maternal FRAA and NT as early prenatal ASD markers.	Giorlandino et al., 2025	11 pregnancies (of 3,600 screened)	[41]	Mechanistic Study
Randomized double-blind placebo-controlled trial: high-dose folinic acid significantly improved verbal communication in children with autism and language impairment, particularly those positive for FRAA.	Frye et al., 2018	48 children (23 folinic, 25 placebo)	[42]	Clinical Trial
EFFET placebo-controlled randomized trial: folinic acid supplementation significantly improved global ADOS autism severity and communication sub-scores versus placebo.	Renard et al., 2020	19 children	[43]	Clinical Trial
Randomized clinical trial in Chinese children with ASD: high-dose folinic acid significantly improved social reciprocity versus TEACCH-only controls, was well tolerated with no significant adverse effects, and MTHFR A1298C / MTRR A66G variant carriers showed greater gains than wild-type.	Zhang, Chen et al., 2025	76 children (49 intervention, 27 control)	[44]	Clinical Trial
Pilot randomized study: folinic acid supplementation in FRAA-positive pregnancies was associated with markedly lower ASD incidence in offspring at 24–30 months versus folic acid (10% vs. 62.5%).	Giorlandino et al., 2026	18 completed (29 randomized, 210 screened)	[45]	Clinical Trial
Systematic review and meta-analysis pooling two double-blind RCTs (Frye 2018; Renard 2020): folinic acid significantly reduced core ASD symptom severity versus placebo.	Soetedjo, Kristijanto & Durry, 2025	103 children (pooled, 2 RCTs)	[46]	Meta-Analysis
Pooled prevalence of ASD in CFD of 44%, CFD in ASD of 38%, FRAA etiology in 83% of CFD-in-ASD cases, and FRAA prevalence in ASD of 71%; children with ASD	Rossignol & Frye, 2021	21 studies pooled	[47]	Meta-Analysis

were >19-fold more likely to test FRAA-positive than typically developing children.				
Clinical review outlining diagnostic work-up and treatment protocols for folate metabolism abnormalities in ASD, including FRAA testing and folinic acid dosing strategy.	Frye et al., 2020	N/A (narrative review)	[48]	Treatment Strategy
Review of FRAA in ASD covering diagnosis, treatment (leucovorin/folinic acid), and prevention strategies; situates FRAA-mediated CFD as a correctable contributor to ASD.	Bobrowski-Khoury, Ramaekers, Sequeira & Quadros, 2021	N/A (narrative review)	[49]	Treatment Strategy
Comprehensive review of CFD syndrome early diagnosis, intervention and treatment strategies, covering intrauterine folate deficiency, hereditary folate malabsorption (PCFT/SLC46A1), and neural tube defect risk.	Ramaekers & Quadros, 2022	N/A (narrative review)	[50]	Treatment Strategy

References

1. Lai, M.; Lee, J.; Chiu, S.; Charm, J.; So, W.Y.; Yuen, F.P.; Kwok, C.; Tsoi, J.; Lin, Y.; Zee, B. A Machine Learning Approach for Retinal Images Analysis as an Objective Screening Method for Children with Autism Spectrum Disorder. *EClinicalMedicine* **2020**, *28*, 100588, doi:10.1016/j.eclinm.2020.100588.
2. Flynn, C.K.; Carr, K.; Whiteley, P.; Nirmalkar, K.; Bellinghiere, A.; Hahn, J.; Liu, H.; Arici, H.; Hewitson, L.; Devlin, M.; et al. Elevated Microbially-Derived Metabolites in Autism: A Possible Diagnostic Screening Test for a Distinct ASD Phenotype. *Mol Psychiatry* **2026**, 1–11, doi:10.1038/s41380-026-03620-5.
3. Tang, X.; Ran, X.; Liang, Z.; Zhuang, H.; Yan, X.; Feng, C.; Qureshi, A.; Gao, Y.; Shen, L. Screening Biomarkers for Autism Spectrum Disorder Using Plasma Proteomics Combined with Machine Learning Methods. *Clin Chim Acta* **2025**, *565*, 120018, doi:10.1016/j.cca.2024.120018.
4. Temiz, M.; Bakir-Gungor, B.; Ersoz, N.S.; Yousef, M. Machine Learning-Based Prediction of Autism Spectrum Disorder and Discovery of Related Metagenomic Biomarkers with Explainable AI. *Applied Sciences* **2025**, *15*, 9214, doi:10.3390/app15169214.
5. Wang, A.; Little, I.D.; Carter, D.; Pham, S.; Piper, M.; Ramírez-Renta, G.M.; Telaak, S.; Gunter, C. Provider-Reported Experiences, Barriers, and Perspectives on Genetic Testing as Part of Autism Diagnosis. *PLOS ONE* **2024**, *19*, e0296942, doi:10.1371/journal.pone.0296942.
6. Briguglio, M.; Turriziani, L.; Currò, A.; Gagliano, A.; Di Rosa, G.; Caccamo, D.; Tonacci, A.; Gangemi, S. A Machine Learning Approach to the Diagnosis of Autism Spectrum Disorder and Multi-Systemic Developmental Disorder Based on Retrospective Data and ADOS-2 Score. *Brain Sci* **2023**, *13*, 883, doi:10.3390/brainsci13060883.

7. Rossignol, D.A.; Frye, R.E. Early Biomarker for Autism Spectrum Disorder Unveiled - What Are We Learning? *Expert Rev Mol Diagn* **2025**, *25*, 413–418, doi:10.1080/14737159.2025.2518143.
8. Huda, S.; Khan, D.M.; Masroor, K.; Warda; Rashid, A.; Shabbir, M. Advancements in Automated Diagnosis of Autism Spectrum Disorder through Deep Learning and Resting-State Functional Mri Biomarkers: A Systematic Review. *Cogn Neurodyn* **2024**, *18*, 3585–3601, doi:10.1007/s11571-024-10176-z.
9. Abdelrahim, M.; Khudri, M.; Elnakib, A.; Shehata, M.; Weafer, K.; Khalil, A.; Saleh, G.A.; Batouty, N.M.; Ghazal, M.; Contractor, S.; et al. AI-Based Non-Invasive Imaging Technologies for Early Autism Spectrum Disorder Diagnosis: A Short Review and Future Directions. *Artif Intell Med* **2025**, *161*, 103074, doi:10.1016/j.artmed.2025.103074.
10. Mertz, L. Using AI and ML to Predict Autism Spectrum Disorder. *IEEE Pulse* **2024**, *15*, 11–15, doi:10.1109/MPULS.2024.3443489.
11. Kim, D.Y.; Do, R.; Shin, Y.; Sim, H.; Kim, H.; Cho, S.; Lee, G.; Park, S.; Jang, B.; Lim, H.; et al. Automated AI Based Identification of Autism Spectrum Disorder from Home Videos. *NPJ Digit Med* **2025**, *8*, 607, doi:10.1038/s41746-025-01993-5.
12. Elbattah, M.; Ali Sadek Ibrahim, O.; Dequen, G. Editorial: Improving Autism Spectrum Disorder Diagnosis Using Machine Learning Techniques. *Front Neuroinform* **2024**, *18*, 1529839, doi:10.3389/fninf.2024.1529839.
13. Tariq, Q.; Daniels, J.; Schwartz, J.N.; Washington, P.; Kalantarian, H.; Wall, D.P. Mobile Detection of Autism through Machine Learning on Home Video: A Development and Prospective Validation Study. *PLOS Medicine* **2018**, *15*, e1002705, doi:10.1371/journal.pmed.1002705.
14. Baran, F.D.E.; Cetin, M. AI-Driven Early Diagnosis of Specific Mental Disorders: A Comprehensive Study. *Cogn Neurodyn* **2025**, *19*, 70, doi:10.1007/s11571-025-10253-x.
15. Pandya, S.; Jain, S.; Verma, J. A Comprehensive Analysis towards Exploring the Promises of AI-Related Approaches in Autism Research. *Comput Biol Med* **2024**, *168*, 107801, doi:10.1016/j.compbiomed.2023.107801.
16. Rajagopalan, S.S.; Zhang, Y.; Yahia, A.; Tammimies, K. Machine Learning Prediction of Autism Spectrum Disorder From a Minimal Set of Medical and Background Information. *JAMA Netw Open* **2024**, *7*, e2429229, doi:10.1001/jamanetworkopen.2024.29229.
17. Wolff, N.; Kohls, G.; Mack, J.T.; Vahid, A.; Elster, E.M.; Stroth, S.; Poustka, L.; Kuepper, C.; Roepke, S.; Kamp-Becker, I.; et al. A Data Driven Machine Learning Approach to Differentiate between Autism Spectrum Disorder and Attention-Deficit/Hyperactivity Disorder Based on the Best-Practice Diagnostic Instruments for Autism. *Sci Rep* **2022**, *12*, 18744, doi:10.1038/s41598-022-21719-x.
18. Leroy, G.; Andrews, J.G.; KeAlohi-Preece, M.; Jaswani, A.; Song, H.; Galindo, M.K.; Rice, S.A. Transparent Deep Learning to Identify Autism Spectrum Disorders (ASD) in EHR Using Clinical Notes. *J Am Med Inform Assoc* **2024**, *31*, 1313–1321, doi:10.1093/jamia/ocae080.
19. Li, Y.; Huang, Y.; Yang, S.; Shychuk, E.M.; Shenkman, E.A.; Bian, J.; Angell, A.M.; Guo, Y. Machine Learning Prediction of Autism Spectrum Disorder Through Linking Mothers' and Children's Electronic Health Record Data. *medRxiv* **2024**, 2024.03.24.24304813, doi:10.1101/2024.03.24.24304813.
20. Mohammadi, F.; Shahrokhi, H.; Asadzadeh, A.; Pirmoradi, S.; Moghtader, A.; Rezaei-Hachesu, P. Artificial Intelligence in Autism Spectrum Disorder Diagnosis: A Scoping Review of Face, Voice, and Text Analysis Methods. *Health Sci Rep* **2025**, *8*, e71476, doi:10.1002/hsr2.71476.
21. Reilly, A.; Walsh, N.; O'Reilly, D.; Smyth, M.; Gorman, K.; Ostadabbas, S.; Power, C. The Role of Machine Learning in Autism Spectrum Disorder Assessment and Management. *Pediatr Res* **2025**, *98*, 2503–2517, doi:10.1038/s41390-025-04566-0.
22. Rajagopalan, S.S.; Ghosh, S. Recent Developments in the Application of Artificial Intelligence and Machine Learning in Early Screening and Diagnosis of Autism. *Methods Mol Biol* **2025**, *2952*, 233–242, doi:10.1007/978-1-0716-4690-8_13.

23. Xu, W.; Li, H.; Li, J.; Jin, M. Age of Machine Learning: New Trends in Autism Spectrum Disorder Prediction. *Front Microbiol* **2025**, *16*, 1492484, doi:10.3389/fmicb.2025.1492484.
24. Aldakhil, A.F.; Alasim, K.N. Diagnostic Accuracy of AI-Based Models for Autism Spectrum Disorder: A Systematic Review and Meta-Analysis with a Focus on Arab Populations. *Res Dev Disabil* **2025**, *167*, 105166, doi:10.1016/j.ridd.2025.105166.
25. Agrawal, R.; Agrawal, R. Explainable AI in Early Autism Detection: A Literature Review of Interpretable Machine Learning Approaches. *Discov Ment Health* **2025**, *5*, 98, doi:10.1007/s44192-025-00232-3.
26. Krishnappa Babu, P.R.; Di Martino, J.M.; Aiello, R.; Eichner, B.; Espinosa, S.; Green, J.; Howard, J.; Perochon, S.; Spanos, M.; Vermeer, S.; et al. Validation of a Mobile App for Remote Autism Screening in Toddlers. *NEJM AI* **2024**, *1*, doi:10.1056/AIcs2400510.
27. Wankhede, N.; Kale, M.; Shukla, M.; Nathiya, D.; R, R.; Kaur, P.; Goyanka, B.; Rahangdale, S.; Taksande, B.; Upaganlawar, A.; et al. Leveraging AI for the Diagnosis and Treatment of Autism Spectrum Disorder: Current Trends and Future Prospects. *Asian J Psychiatr* **2024**, *101*, 104241, doi:10.1016/j.ajp.2024.104241.
28. 2016 International Meeting for Autism Research: Use of Machine Learning for Behavioral Distinction of Autism and ADHD Available online: https://imfar.confex.com/imfar/2016/webprogram/Paper22933.html (accessed on 7 July 2026).
29. Rakotomanana, H.; Rouhafzay, G. A Scoping Review of AI-Based Approaches for Detecting Autism Traits Using Voice and Behavioral Data. *Bioengineering (Basel)* **2025**, *12*, 1136, doi:10.3390/bioengineering12111136.
30. Eslami, T.; Almuqhim, F.; Raiker, J.S.; Saeed, F. Machine Learning Methods for Diagnosing Autism Spectrum Disorder and Attention- Deficit/Hyperactivity Disorder Using Functional and Structural MRI: A Survey. *Front Neuroinform* **2020**, *14*, 575999, doi:10.3389/fninf.2020.575999.
31. Alsharif, N.; Al-Nefaie, A.H.; Ahmad, S.; Farhah, N.S. Artificial Intelligence-Driven Diagnosis of Autism Spectrum Disorder in Children. *Front Med (Lausanne)* **2025**, *12*, 1723320, doi:10.3389/fmed.2025.1723320.
32. Demetriou, E.A.; Park, S.H.; Ho, N.; Pepper, K.L.; Song, Y.J.C.; Naismith, S.L.; Thomas, E.E.; Hickie, I.B.; Guastella, A.J. Machine Learning for Differential Diagnosis Between Clinical Conditions With Social Difficulty: Autism Spectrum Disorder, Early Psychosis, and Social Anxiety Disorder. *Front Psychiatry* **2020**, *11*, 545, doi:10.3389/fpsyt.2020.00545.
33. Ramaekers, V.T.; Häusler, M.; Opladen, T.; Heimann, G.; Blau, N. Psychomotor Retardation, Spastic Paraplegia, Cerebellar Ataxia and Dyskinesia Associated with Low 5-Methyltetrahydrofolate in Cerebrospinal Fluid: A Novel Neurometabolic Condition Responding to Folinic Acid Substitution. *Neuropediatrics* **2002**, *33*, 301–308, doi:10.1055/s-2002-37082.
34. Ramaekers, V.T.; Blau, N. Cerebral Folate Deficiency. *Dev Med Child Neurol* **2004**, *46*, 843–851, doi:10.1017/s0012162204001471.
35. Ramaekers, V.T.; Sequeira, J.M.; Blau, N.; Quadros, E.V. A Milk-Free Diet Downregulates Folate Receptor Autoimmunity in Cerebral Folate Deficiency Syndrome. *Dev Med Child Neurol* **2008**, *50*, 346–352, doi:10.1111/j.1469-8749.2008.02053.x.
36. Frye, R.E.; Sequeira, J.M.; Quadros, E.V.; James, S.J.; Rossignol, D.A. Cerebral Folate Receptor Autoantibodies in Autism Spectrum Disorder. *Mol Psychiatry* **2013**, *18*, 369–381, doi:10.1038/mp.2011.175.
37. Frye, R.E.; Delhey, L.; Slattery, J.; Tippett, M.; Wynne, R.; Rose, S.; Kahler, S.G.; Bennuri, S.C.; Melnyk, S.; Sequeira, J.M.; et al. Blocking and Binding Folate Receptor Alpha Autoantibodies Identify Novel Autism Spectrum Disorder Subgroups. *Frontiers in Neuroscience* **2016**, *10*.
38. Frye, R.E.; Slattery, J.C.; Quadros, E.V. Folate Metabolism Abnormalities in Autism: Potential Biomarkers. *Biomarkers in Medicine* **2017**, *11*, 687–699, doi:10.2217/bmm-2017-0109.

39. Kakkassery, V.; Koschmieder, A.; Walther, F.; Lehbrink, R.; Bertsche, A.; Wortmann, S.B.; Buchmann, J.; Jäger, M.; Friedburg, C.; Lorenz, B.; et al. [Chorioretinal atrophy in pediatric cerebral folate deficiency-a preventable disease?]. *Ophthalmologe* **2021**, *118*, 383–390, doi:10.1007/s00347-020-01126-1.
40. Gurler, G.; Belder, N.; Beker, M.C.; Sever-Bahcekapili, M.; Uruk, G.; Kilic, E.; Yemisci, M. Reduced Folate Carrier 1 Is Present in Retinal Microvessels and Crucial for the Inner Blood Retinal Barrier Integrity. *Fluids Barriers CNS* **2023**, *20*, 47, doi:10.1186/s12987-023-00442-3.
41. Giorlandino, C.; Margiotti, K.; Fabiani, M.; Mesoraca, A.; D'Emidio, L.; Raffio, R.; Coco, C.; Mastrandrea, M.L.; Pasquale, C.; Cupellaro, M.; et al. Maternal Folate Receptor Alpha Autoantibodies and Increased Fetal Nuchal Translucency as Potential Early Markers of Autism Spectrum Disorder. *Brain and Behavior* **2025**, *15*, e71088, doi:10.1002/brb3.71088.
42. Frye, R.E.; Slattery, J.; Delhey, L.; Furgerson, B.; Strickland, T.; Tippett, M.; Sailey, A.; Wynne, R.; Rose, S.; Melnyk, S.; et al. Folinic Acid Improves Verbal Communication in Children with Autism and Language Impairment: A Randomized Double-Blind Placebo-Controlled Trial. *Mol Psychiatry* **2018**, *23*, 247–256, doi:10.1038/mp.2016.168.
43. Renard, E.; Leheup, B.; Guéant-Rodriguez, R.-M.; Oussalah, A.; Quadros, E.V.; Guéant, J.-L. Folinic Acid Improves the Score of Autism in the EFFET Placebo-Controlled Randomized Trial. *Biochimie* **2020**, *173*, 57–61, doi:10.1016/j.biochi.2020.04.019.
44. Zhang, C.; Chen, Y.; Hou, F.; Li, Y.; Wang, W.; Guo, L.; Zhang, C.; Li, L.; Lu, C. Safety and Efficacy of High-Dose Folinic Acid in Children with Autism: The Impact of Folate Metabolism Gene Polymorphisms. *Nutrients* **2025**, *17*, 1602, doi:10.3390/nu17091602.
45. Giorlandino, C.; Mesoraca, A.; Margiotti, K.; Fabiani, M.; Cupellaro, M.; Giorlandino, F.; Mastrandrea, M.L.; Raffio, R.; Pignataro, F.; Mangiafico, L.; et al. Folinic Acid Supplementation in Folate Receptor Alpha Autoantibodies-Positive Pregnancy: A Pilot Randomized Study on Neurodevelopmental Outcomes. *Reproductive, Female and Child Health* **2026**, *5*, e70053, doi:10.1002/rfc2.70053.
46. Soetedjo, F.; Kristijanto, J.A.; Durry, F. Folinic Acid and Autism Spectrum Disorder in Children: A Systematic Review and Meta-Analysis of Two Double-Blind Randomized Placebo-Controlled Trials. *AcTion: Aceh Nutrition Journal* **2025**, *10*, 194, doi:10.30867/action.v10i1.1743.
47. Rossignol, D.A.; Frye, R.E. Cerebral Folate Deficiency, Folate Receptor Alpha Autoantibodies and Leucovorin (Folinic Acid) Treatment in Autism Spectrum Disorders: A Systematic Review and Meta-Analysis. *JPM* **2021**, *11*, 1141, doi:10.3390/jpm11111141.
48. Frye, R.E. Mitochondrial Dysfunction in Autism Spectrum Disorder: Unique Abnormalities and Targeted Treatments. *Seminars in Pediatric Neurology* **2020**, *35*, 100829, doi:10.1016/j.spen.2020.100829.
49. Bobrowski-Khoury, N.; Ramaekers, V.T.; Sequeira, J.M.; Quadros, E.V. Folate Receptor Alpha Autoantibodies in Autism Spectrum Disorders: Diagnosis, Treatment and Prevention. *JPM* **2021**, *11*, 710, doi:10.3390/jpm11080710.
50. Ramaekers, V.Th.; Quadros, E.V. Cerebral Folate Deficiency Syndrome: Early Diagnosis, Intervention and Treatment Strategies. *Nutrients* **2022**, *14*, 3096, doi:10.3390/nu14153096.

Disclaimer/Publisher's Note: The statements, opinions and data contained in all publications are solely those of the individual author(s) and contributor(s) and not of MDPI and/or the editor(s). MDPI and/or the editor(s) disclaim responsibility for any injury to people or property resulting from any ideas, methods, instructions or products referred to in the content.
